# Evaluation of GBLUP, BayesB and elastic net for genomic prediction in Chinese Simmental beef cattle

**DOI:** 10.1371/journal.pone.0210442

**Published:** 2019-02-28

**Authors:** Xiaoqiao Wang, Jian Miao, Tianpeng Chang, Jiangwei Xia, Binxin An, Yan Li, Lingyang Xu, Lupei Zhang, Xue Gao, Junya Li, Huijiang Gao

**Affiliations:** 1 Laboratory of Molecular Biology and Bovine Breeding, Institute of Animal Sciences, Chinese Academy of Agricultural Sciences, Beijing, China; 2 Veterinary Bureau of Wulagai Precinct in Xilin Gol League, Wulagai, China; The University of Sydney, AUSTRALIA

## Abstract

Chinese Simmental beef cattle are the most economically important cattle breed in China. Estimated breeding values for growth, carcass, and meat quality traits are commonly used as selection criteria in animal breeding. The objective of this study was to evaluate the accuracy of alternative statistical methods for the estimation of genomic breeding values. Analyses of the accuracy of genomic best linear unbiased prediction (GBLUP), BayesB, and elastic net (EN) were performed with an Illumina BovineHD BeadChip on 1,217 animals by applying 5-fold cross-validation. Overall, the accuracies ranged from 0.17 to 0.296 for ten traits, and the heritability estimates ranged from 0.36 to 0.63. The EN (alpha = 0.001) model provided the most accurate prediction, which was also slightly higher (0.2–2%) than that of GBLUP for most traits, such as average daily weight gain (ADG) and carcass weight (CW). BayesB was less accurate for each trait than were EN (alpha = 0.001) and GBLUP. These findings indicate the importance of using an appropriate variable selection method for the genomic selection of traits and suggest the influence of the genetic architecture of the traits we analyzed.

## Introduction

Chinese Simmental beef cattle play an important role in the Chinese beef industry because of their high adaptability and the rapid growth of their young with sufficient feeding. The beef is also well marbled and tender, contributing to its marketability. The primary objective of beef production is to improve beef quality and yield grades. Estimated breeding values of economical traits, such as carcass weight (CW), eye muscle area (EMA), and marbling score (MS), are generally considered to be the major selection criteria in beef cattle breeding. The average daily weight gain (ADG) is also important as an indicator of growth.

In China, small holders manage a large proportion of young bulls, in some cases keeping fewer than 100 cattle. Considering this situation, these bulls cannot be managed well. The bulls often lack phenotypic or pedigree records due to the absence of unified management. In this context, an alternative to traditional genetic evaluations is genomic selection (GS), which was originally introduced by [1]. GS is the selection for economic quantitative traits in animals and plants based on their genome-wide estimated breeding values (GEBVs), which are calculated based on dense markers and do not depend on pedigree information. With the availability of high-throughput dense genotyping of SNPs and several statistical models, GS is currently being adopted in animal breeding. The predictive accuracy is key to the success of genomic prediction. However, no consensus exists on the best approach, which is dependent on the genetic architecture of traits.

The methods for genomic prediction can be classified into two groups: linear and nonlinear methods. The commonly used linear methods include unbiased and biased prediction models. Unbiased prediction models include genomic best linear unbiased prediction (GBLUP) and ridge regression best linear unbiased prediction (RR-BLUP) [2], while biased prediction models are also known as regularized (penalized) linear regression models such as ridge regression (RR) [3], the least absolute shrinkage and selection operator (LASSO) [4], and elastic net (EN) [5]. Bayesian methods are nonlinear methods and include Bayes A/B/C/Cπ/R. They are often implemented by MCMC (Markov chain Monte Carlo) sampling to obtain the variable of shrinkage coverage. GBLUP is a routine genetic evaluation for livestock because it is simple and less computationally demanding. RR-BLUP has been found to be equivalent to GBLUP procedures [6]. The EN with alpha, an adjustable parameter, set to 0 is equivalent to RR, and with alpha close to 1 performs very similarly to LASSO. Moreover, EN produces more correctly identified influential variables than LASSO and has a much lower false positive rate than RR [7].

Previous studies have reported the performance of genomic prediction methods for various traits in different breeds. These studies suggested that statistical methods may perform differently for different traits because of the large differences in the genetic architecture of complex traits [8]. When fewer genes with large effects influence traits, Bayesian models have a small advantage over linear models such as GBLUP, whereas GBLUP may outperform BayesB for a trait with many loci with small effects. LASSO and EN approaches can compress the small size effect to zero with a penalty based on functions of magnitude of effect for each SNP [9]. Previous studies have investigated the application of Bayesian methods for GS with several traits in Chinese Simmental beef cattle [10,11]. However, the GS of bone weight (BNW), MS and carcass length (CL) has not been reported. Moreover, the accuracy of GS using regularized linear regression models for many growth, carcass, and meat quality traits in Chinese Simmental beef cattle and even other breeds of beef cattle remains largely unknown, particularly the accuracy of the EN method. When setting an appropriate alpha in an EN model, the results may be better than those of Bayesian methods and GBLUP.

The aim of this study was to provide a scientific basis for the application of GS to the Chinese Simmental population. A comparative evaluation of the performance of multiple methods is essential to identify those best suited to GS for Chinese Simmental cattle. Here, we evaluated the relative performance of five methods for GS. We expected to identify suitable methods for estimating the predictive accuracy for various growth, carcass and meat quality traits of economic relevance using the BovineHD SNP array in the Chinese Simmental cattle population.

## Materials and methods

### Ethics statement

All animals used in the study were treated following the guidelines established by the Council of China Animal Welfare. The research was undertaken with the approval of the Committee on the Ethics of Animal Experiments of the Chinese Academy of Agricultural Sciences (CAAS) (Beijing, China). The use of animals and private land in this study was approved by the respective owners.

### Animal and phenotypic data

Our resource population included 1,225 Chinese Simmental cattle born between 2008 and 2014 in Ulgai, Xilingol League and Inner Mongolia of China. After weaning, these cattle were moved to Jinweifuren Co., Ltd. for fattening, with all animals sharing uniform feeding and management conditions. More detailed descriptions of the breeding and management have been described previously [12]. The cattle were slaughtered at an average age of 16–18 months. At slaughter, the carcass trait and meat quality traits were assessed according to the Institutional Meat Purchase Specifications for Fresh Beef Guidelines. Traits in this study were the following: ADG (kg/d), live weight (LW, kg), CW (kg), BNW (kg), tenderloin weight (TW, kg), sirloin weight (SW, kg), EMA (cm^2^), CL (cm), hind leg length (HLL, cm), and MS. The ADG was the rate of weight gain per day over the fattening duration. LW was measured before slaughter after fasting for 24 h. CW was the remaining cold carcass measured after slaughter and bloodletting by eliminating the hide, head, feet, tail, entrails and gut fill. The total BNW was created to sum the weights of the carcass bones. EMA was measured at the 12th and 13th rib interface 48 h postmortem via the dot grid method. TW, SW and CL were measured directly from carcass anatomy. MS was visually scored on a seven-point scale depending on the degree of marbling on the cut surface of the rib eye. The statistics used for each trait to estimate variance components are presented in Table 1.

**Table 1:** Descriptive statistics of phenotypic data used in the genomic prediction.

Trait (unit)	N^a^	*h*^*2*^ (SE)	Mean (SE)	Min.	Max.	SD
ADG (kg)	1,216	0.44 ± 0.07	0.97 ± 0.01	0.38	2.41	0.22
LW (kg)	1,216	0.53 ± 0.07	505.26 ± 2.03	318.00	776.00	70.76
CW (kg)	1,216	0.59 ± 0.07	271.35 ± 1.31	162.60	486.00	45.65
BNW (kg)	1,214	0.60 ± 0.07	40.67 ± 0.19	20.20	80.00	6.52
SW (kg)	1,213	0.45 ± 0.07	8.67 ± 0.06	3.21	15.90	1.96
TW (kg)	1,215	0.63 ± 0.06	3.98 ± 0.71	2.20	7.84	0.71
EMA (cm^2^)	1,117	0.57 ± 0.07	85.21 ± 0.4	51.00	150.00	13.32
CL (cm)	1,212	0.44 ± 0.08	138.36 ± 0.20	115.00	164.00	6.91
HLL (cm)	1,214	0.52 ± 0.07	76.88 ± 0.15	50.00	92.00	5.24
MS (cm^2^)	1,214	0.36 ± 0.08	5.130 ± 0.03	1.00	7.00	0.97

*h*^*2*^ heritability, SE standard error, ADG average daily weight gain, LW live weight, CW carcass weight, BNW bone weight, SW sirloin weight, TW tenderloin weight, EMA eye muscle area, CL carcass length, HLL hand legs length, MS marbling score

^a^Number of animal with phenotypes

The fixed effects were used to adjust the phenotypic values of traits of interest before the analysis:

$$y=X\beta +y^{*}$$

where y is a vector of observed phenotypic values, *β* is a vector of fixed effects (year of birth and sex as a contemporary group; fattening duration and initial body weight as a covariate), *X* is the design matrix of relevant observations to the corresponding fixed effects, and y* is the random residual. The residual y* was subsequently used in the prediction models.

### Genotyping and quality control

Genotypes were generated with the Illumina BovineHD BeadChip. Quality control of genotypes was conducted through an iterative process using the following SNP selection criteria: call rate (CR) higher than 0.95, minor allele frequency (MAF) higher than 0.05 and *P*-value for Hardy-Weinberg equilibrium test (HWE) higher than 10^−5^. The samples with more than 10% missing genotypes were excluded, which resulted in a final dataset of 1,217 cattle with 608,696 SNPs. Additionally, the imputation for sporadic missing alleles was performed using Beagle 3.3.2 [13].

### Genomic prediction methods

GBLUP and BayesB: GEBVs were calculated based on the following equation:

$$y^{*}=1\mu +Z\gamma +e$$ (1)

where y* is the vector of the corrected phenotype, *μ* is the overall mean, and $e\sim {N(0,I\sigma }_{e}^{2})$ is a vector of residual error, where $\sigma_{e}^{2}$ is the residual variance. *I* is an identity matrix. For GBLUP, *Z* is an incidence matrix for individual effects. *γ* is the vector of breeding values assumed to follow a multivariate normal distribution $\text{MVN}\sim N(0,\;K\sigma_{g}^{2})$, where $\sigma_{g}^{2}$ is genetic variance, *K* is calculated following $K=\frac{1}{d}\sum_{k=1}^{m}Z_{k}Z_{k}^{T}$ [14], and $d=\frac{1}{n}tr(\sum_{k=1}^{m}Z_{k}Z_{k}^{T})$. *Z*_*k*_ is the vector of SNP genotypes for *n* individuals at locus *k* for *k* = 1, …, *m*, where *m* is the number of markers and *n* is the number of individuals. SNP genotypes are represented as 1, 0, or -1 to denote a diploid genotype value of 11, 12, or 22, respectively. For BayesB, *Z* is a genotype matrix corresponding to *γ*. $\gamma \sim N(0,\sigma_{gk}^{2})$ is the vector of SNP effects. The variance of the *k*^*th*^ SNP effect, $\sigma_{gk}^{2}$, is assigned an informative prior to show the presence (with probability 1 − *π*) and absence (with probability *π*) of the marker *k*.

Elastic net method: y* is treated as the corrected phenotype and is described by the following multiple regression model:

$$y^{*}=Z\gamma +e=\sum_{k=1}^{m}Z_{k}\gamma_{k}+e$$ (2)

where y* is the vector of the corrected phenotype. *Z* is the matrix of genotype codes for SNPs with -1, 0, and 1. *γ* is the vector of SNP effects. $e\sim N(0,I\sigma_{e}^{2})$ is the vector of residual error. *Z*_*k*_ is the vector of SNP genotypes for *n* individuals at locus *k* for *k* = 1, …, *m*, where *m* is the number of markers and *n* is the number of individuals. *γ*_*k*_ is the SNP effects for marker *k*. The ordinary least squares (OLS) estimates the parameter *γ* by minimizing the residual sum of squares:

$$\gamma^{RSS}=\text{arg}\;{\text{min}}_{\gamma \in \Omega }\sum_{i=1}^{n}{\left(y^{*}-\sum_{k=1}^{m}Z_{k}\gamma_{k}\right)}^{2}$$ (3)

The LASSO [15] penalty estimates marker effects via adding an L1-norm in the OLS:

$$\gamma^{LASSO}=\text{arg}\;{\text{min}}_{\gamma \in \Omega }\left\{\sum_{i=1}^{n}{\left(y^{*}-\sum_{k=1}^{m}Z_{k}\gamma_{k}\right)}^{2}+\lambda \sum_{k=1}^{m}\left|\gamma_{k}\right|\right\}$$ (4)

The RR [3] penalty estimates marker effects via adding an L2-norm in the OLS:

$$\gamma^{RR}=\text{arg}\;{\text{min}}_{\gamma \in \Omega }\left\{\sum_{i=1}^{n}{\left(y^{*}-\sum_{k=1}^{m}Z_{k}\gamma_{k}\right)}^{2}+\lambda \sum_{k=1}^{m}\gamma_{k}^{2}\right\}$$ (5)

The EN [5] penalty estimates marker effects via adding a synthetic of L1-norm and L2-norm in the OLS. Thus, EN is based on a compromise between LASSO and RR, where alpha is for the EN mixing parameter (0 ≤ α ≤ 1):

$$\gamma^{Elastic\;net}=\text{arg}\;{\text{min}}_{\gamma \in \Omega }\left\{\sum_{i=1}^{n}{\left(y^{*}-\sum_{k=1}^{m}Z_{k}\gamma_{k}\right)}^{2}+\lambda \sum_{k=1}^{m}\left(\frac{\left(1-\alpha \right)}{2}\gamma_{k}^{2}+\alpha |\gamma_{k}|\right)\right\}$$ (6)

The lambda in Eqs (4), (5) and (6) is a regularization parameter that controls the amount of shrinkage. The optimal value of λ can then be found by k-fold cross-validation (CV) to identify the minimum mean squared error (minMSE).

All analyses were conducted using R language; GBLUP was obtained by solving the mixed model equations (MMEs). The BGLR package was used to conduct BayesB. RR, LASSO, and EN were conducted in the glmnet R package. For BayesB, the MCMC was run for 50,000 iterations with a burn in of 2,500 and thinning of 10. These numbers of iterations were sufficient in that increasing the number did not change the results. An MCMC sampler with shorter chain and burn-in (22,000 iterations and 2,500 burn in) was tested, but the results were discarded. To identify the best performance of the EN method, we used penalty weights of α = 0.001, 0.01, 0.05, 0.1, 0.4, 0.7, and 1.

### Estimation of genetic parameters

Genomic heritability, genetic correlation, and phenotypic correlation were analyzed using the ASReml v3.0 software package [16]. An animal model was applied to estimate the heritability of all traits. A pairwise bivariate animal model was applied to estimate the genetic correlation between both traits. The model is

[y1y2]=[X100X2][b1b2]+[Z100Z2][a1a2]+[e1e2] (7)

where *y*_1_ and *y*_2_ are vectors of trait 1 and 2, respectively; *X*_1_ and *X*_2_ are incidence matrices for fixed effects; *b*_1_ and *b*_2_ are the vctors of the fixed effects; *Z*_1_ and *Z*_2_ are incidence matrices relating the phenotypic observations to vectors of the polygenic effects for two traits; and *e*_1_ and *e*_2_ are random residuals for two traits.

For GBLUP, the GEBVs of all genotyped individuals were predicted by solving the MMEs. For BayesB and EN methods, the GEBV was calculated by adding all the marker effects estimated from the training population. The formula was $GEBV=\sum_{k=1}^{m}Z_{k}\gamma_{k}$.

### Validation population

Two validation procedures were considered for assessing the predictive accuracy of different methods in this article. We mainly used 5-fold CV procedures. The other method is a generation validation procedure. Because phenotype records were not available for all genotyped animals for all traits, the number of animals in the training and test datasets differed among traits. However, the test populations were the same for each method. In 5-fold CV procedures, the population (for each trait) was randomly split into five parts of approximately equal size. Then, the analysis was performed using each subset of the data as the validation sample and the other 4 subsets as the training population. For EMA, the numbers of animals in training and test populations were 893 and 224, respectively. For the other traits, the sizes of the training and validation populations depended on the traits.

In generation validation procedures, the dataset was divided into training (animals from 2008 to 2013) and test (animals from 2014) subgroups, which contained 1135 and 82 animals, respectively. Three traits were measured in this part, including ADG, CW and MS. The accuracy of the genomic predictions was measured as the Pearson correlation between y* and predicted GEBV on the test subgroup using the formula $r(y_{test}^{*}{,GEBV}_{test})=\frac{cov(y_{test}^{*},{GEBV}_{test})}{\sqrt{var(y_{test}^{*})var({GEBV}_{test})}}$. Realized accuracy was calculated as $\frac{r(y_{test}^{*}{,GEBV}_{test})}{\sqrt{trait\;heritability}}$ [17].

## Results

### Descriptive statistics and genetic parameters

**Fig 1:**
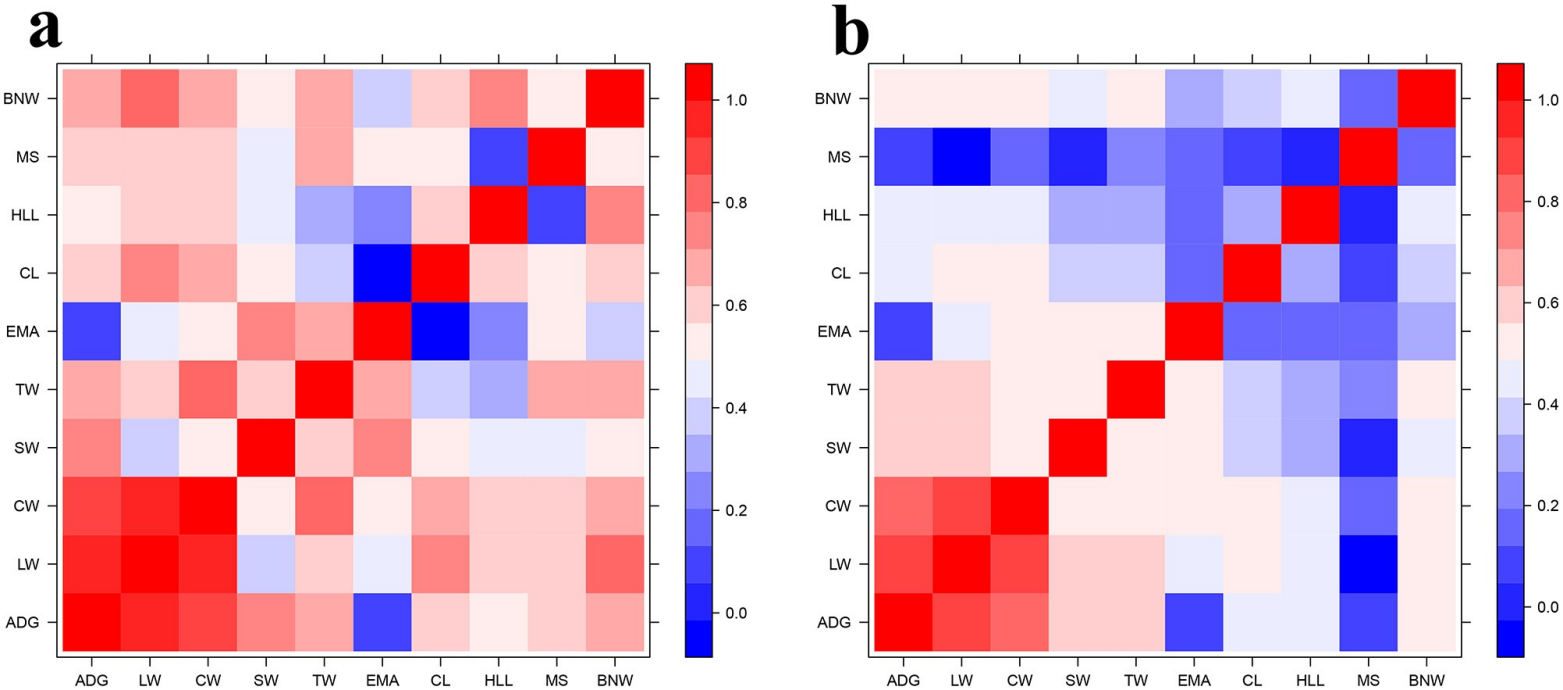
Heat map of phenotypic (a) and genetic correlation (b) across ten traits.

The estimates of heritability for ten traits are presented in Table 1. Of the ten traits, the one growth trait, ADG, had a median heritability of 0.44. Eight traits were carcass traits, including LW, CW, BNW, SW, TW, EMA, CL and HLL, and most had high heritability that ranged from 0.57 for EMA to 0.63 for TW. As a meat quality trait, MS had moderate heritability (0.36). The heritabilities of the traits ADG, CW, EMA, and MS for other breeds of beef cattle are shown in Table 2. The estimated heritabilities for CW (0.59) and TW (0.63) were higher (+0.14) than those previously reported in Chinese Simmental beef cattle, but the heritability of CW was similar to that of Japanese Black cattle [18]. Heritabilities for traits ADG (0.44) and EMA (0.57) were consistent with previous reports for Chinese Simmental beef cattle (0.47) and American Angus cattle (0.51), respectively. Heritability for trait MS (0.36) was below the range of 0.4–0.69 reported in previous studies. The reason for such large differences in heritability is partially the different populations and different marker-based relationship matrices used for heritability estimations. The phenotypic and genetic correlations among the 10 traits are shown in Fig 1 and S1 Table. ADG had low genetic and phenotypic correlations with both EMA and MS. Carcass traits had moderate, even high, genetic and phenotypic correlation with one another in most cases. EMA had low genetic and phenotypic correlations with both ADG, CL and HLL. MS also had the lowest phenotypic correlations, ranging from -0.05 for LW to 0.24 for TW, although MS had a moderate genetic correlation with most traits. Additionally, a heat map was used to visualize the kinship among individuals (S1 Fig).

**Table 2:** Summary of the genomic prediction accuracy and heritability of five traits in beef cattle from different countries.

Beef cattle (N^a^)	Traits	Heritability	Prediction accuracy ^b^	Reference
Chinese Simmental (1,302)	ADG	0.47	0.214 (GBLUP)	[10]
	CW	0.45	0.285 (PBayesB)	
Chinese Simmental (1,173)	TW	0.47	0.566^c^ (BayesB)	[11]
	CW	0.38	0.487^c^ (BayesB)	
Hanwoo (1,183)	CW	0.33	0.4 (BayesC)	[25]
	EMA	0.37	0.317 (BayesC)	
	MS	0.4–0.42	0.25 (BayesL)	
Nellore (1,756)	CW	0.17	0.37^c^ (BayesLasso)	[30]
	EMA	0.20	0.47^c^ (BayesLasso)	
Nelore (803)	ADG	0.31,0.53,0.41	0.26 (BGBLUP, BayesA, BayesCπ)	[31]
American Angus (3,570)	CW	0.40	0.689^d^ (BayesC)	[26]
	EMA	0.51	0.698^d^ (BayesC)	
	MS	0.45	0.817^d^ (BayesC)	
Japanese Black (20,436)	CW	0.56	0.44 (ssGBLUP, *τ*^3^ = 1)	[18]
	EMA	0.42	0.42 (ssGBLUP, *τ*^3^ = 1)	
	MS	0.69	0.39 (ssGBLUP, *τ*^3^ = 0.5)	
Multibreed (6,796)	ADG	0.38	0.36^c^ (BayesC)	[32]

Trait: ADG average daily gain, CW carcass weight, TW tenderloin weight, EMA eye muscle area, MS marbling score

Methods: PBayesB Parallel BayesB, BGBLUP Bayesian GBLUP, ssGBLUP single step GBLUP

^a^ Number of animals with phenotypes

^b^ the highest empirical prediction accuracies in documented findings

^c^ Realized accuracy: the prediction accuracy was divided by the square root of heritability of the trait

^d^ The accuracy could be defined as the correlation between true genetic values and directly genomic values (DGV) divided by the square root of heritability of the traits

### Accuracy of GEBV with LASSO and EN models in 5-fold CV

Table 3 shows the accuracy of genomic prediction using the EN method for the ten traits. All traits were evaluated at the following penalty weights: α = 1, 0.7, 0.4, 0.1, 0.05, 0.01 and 0.001, where α = 1 is equivalent to LASSO. We also performed RR with α = 0, but the results were poor and included some NA, which might have been caused by nonconvergence. Thus, Table 3 does not show the results of the RR method. There is a tendency for higher accuracies to appear in the EN model with a smaller alpha setting. In particular, when setting alpha = 0.001, the results were generally better than the others, except for the trait BNW, which performed better with an α = 0.1. Thus, the EN method (α = 0.001) was used to compare with the GBLUP and BayesB.

**Table 3:** Accuracies of genomic EBV of 5-fold cross-validation population using regularized regression methods for ten traits.

Trait	EN(0.001)	EN(0.01)	EN(0.05)	EN(0.1)	EN(0.4)	EN(0.7)	EN(1)
ADG	0.251	0.244	0.221	0.221	0.191	0.186	0.185
LW	0.290	0.290	0.284	0.277	0.266	0.263	0.263
CW	0.292	0.278	0.265	0.259	0.246	0.243	0.241
BNW	0.295	0.308	0.312	0.311	0.305	0.302	0.301
SW	0.259	0.249	0.235	0.229	0.210	0.204	0.201
TW	0.296	0.299	0.297	0.293	0.279	0.276	0.274
EMA	0.281	0.263	0.251	0.248	0.243	0.240	0.238
CL	0.181	0.169	0.151	0.141	0.121	0.125	0.114
HLL	0.274	0.272	0.259	0.252	0.245	0.241	0.240
MS	0.180	0.159	0.133	0.121	0.094	0.088	0.087

EN elastic net method, ADG average daily weight gain, LW live weight, CW carcass weight, BNW bone weight, SW sirloin weight, TW tenderloin weight, EMA eye muscle area, CL carcass length, HLL hand legs length, MS marbling score

### Accuracy of GEBV in the GBLUP, EN and BayesB models in 5-fold CV

Table 4 shows the genomic prediction accuracy using different methods for the ten traits and their coefficients of variation (*C*_*v*_). The average accuracy of the GEBV for the ten traits was approximately 0.25, ranging from 0.18 to 0.30. Among the three methods, the lowest GEBV accuracy was found in BayesB for each trait. The prediction method yielding the highest accuracy differed among traits. GBLUP had the highest accuracy for traits EMA, CL, and MS, whereas EN had the highest accuracy for traits ADG, LW, BNW, SW, TW, and HLL. The comparable performances of the GBLUP, BayesB and EN methods suggested that the accuracy of the EN method (α = 0.001) was slightly better (0.1 to 2.5%) than that of the GBLUP and BayesB methods for six traits. GBLUP usually performed well, whereas the BayesB method performed poorly. The *C*_*v*_s were similar among the methods, except for trait BNW, which was higher (+0.07) in the EN method than that of the other two methods. Additionally, we computed the accuracy of the GEBV using the RR-BLUP method, and the results were the same as those using GBLUP for each trait; therefore, the results are not shown in this study. We also fixed π = 0.99 and 0.999 in the BayesB method for the CW, ADG, EMA, and MS traits (Table 5).

**Table 4:** Comparison of the genomic prediction accuracy (Acc) and coefficient variation (*C*_*v*_) for ten traits using three methods.

Trait	GBLUP		EN (0.001)		BayesB (π = 0.9)	
	Acc	*C*_*v*_	Acc	*C*_*v*_	Acc	*C*_*v*_
ADG	0.243	0.20	0.251	0.20	0.239	0.19
LW	0.275	0.19	0.290	0.19	0.265	0.20
CW	0.290	0.17	0.292	0.17	0.282	0.18
BNW	0.295	0.18	0.295	0.25	0.294	0.19
SW	0.241	0.21	0.259	0.19	0.234	0.23
TW	0.294	0.17	0.296	0.16	0.287	0.16
EMA	0.287	0.19	0.281	0.20	0.281	0.18
CL	0.184	0.32	0.181	0.33	0.177	0.34
HLL	0.254	0.17	0.274	0.17	0.246	0.19
MS	0.184	0.29	0.180	0.28	0.171	0.32

Trait: ADG average daily weight gain, LW live weight, CW carcass weight, BNW bone weight, SW sirloin weight, TW tenderloin weight, EMA eye muscle area, CL carcass length, HLL hand legs length, MS marbling score

Method: GBLUP genomic best linear unbiased prediction, EN elastic net method

**Table 5:** Accuracies of genomic EBV of 5-fold cross-validation population using BayesB method for two traits.

Trait	BayesB		
	π = 0.999	π = 0.99	π = 0.9
ADG	0.095	0.206	0.239
CW	0.165	0.260	0.282
EMA	0.149	0.241	0.281
MS	0.044	0.137	0.171

ADG average daily weight gain, CW carcass weight, EMA eye muscle area, MS marbling score

### Accuracy of GEBV in the GBLUP, EN and BayesB models in generation validation

Table 6 shows the genomic prediction accuracy using different methods for the trait ADG, CW, and MS in generation validation. The EN method (α = 0.0001) performed the best among the three methods for each trait. For ADG, the BayesB method had the lowest accuracy, of 0.243 compared to 0.261 for EN and 0.250 for GBLUP. For CW, the three methods had similar results, at approximately 0.31. The accuracy of these two traits using GBLUP was slightly lower (0.004, 0.01 respectively) than that using the EN method. GBLUP had the lowest accuracy, 0.186, for measuring MS.

**Table 6:** Accuracies of genomic EBV of generation validation population for three traits using three method.

Trait	GBLUP	EN (0.0001)	BayesB (π = 0.9)
ADG	0.250	0.261	0.243
CW	0.311	0.315	0.312
MS	0.186	0.191	0.190

Trait: ADG average daily weight gain, CW carcass weight, MS marbling score

Method: GBLUP genomic best linear unbiased prediction, EN elastic net method

## Discussion

The primary aim of this study was to compare the prediction ability of different methods and identify a suitable GS model for the most economical traits in Chinese Simmental cattle. Based on the empirical prediction accuracy results, EN (α = 0.001) and GBLUP outperformed BayesB. Compared with those of the GBLUP and BayesB methods, EN (α = 0.001) predictions had higher accuracy for most traits. LASSO had the lowest accuracy for most traits. In a previous study on genomic prediction, most cases showed that the accuracy of GBLUP outperformed that of the Bayesian method with real data, with the opposite trend for simulation data [19,20].

### Comparison of regularized linear regression models to estimate GEBV in 5-fold CV

EN, LASSO and RR are penalized least-squares methods. We used the glmnet/R package to perform EN, LASSO and RR analyses because glmnet/R is extremely fast for use with our dataset. In glmnet/R, the optimal value of the tuning parameter λ, which controls the degree of shrinkage, can be obtained through CV. The λ value producing the minMSE is deemed the optimal tuning parameter in training sets. To avoid the overfitting problem in training sets, some scientists advise using minMSE+1SE to obtain the optimal λ [21]. We tested the minMSE+1SE stopping criterion, but the results were not as good as those using minMSE.

RR assumes that many predictors all have nonzero coefficients and that they obey a normal distribution [22]. Specifically, the performance is good when there are many predictors, each of which has a small effect. However, in our investigation, the performance of RR was unsatisfactory. A trend occurred in which higher accuracy values appeared in the EN model with a smaller alpha setting. Specifically, when the setting α = 0.001, the results were generally better than those of the other scenarios, particularly the LASSO method (α = 0). For the trait BNW, EN with α = 0.1 performed the best. These results are consistent with those of a previous study in which EN outperformed LASSO [23]. The regularization of LASSO results in many regression coefficients trending toward zero; therefore, LASSO as an automatic variable selection method could select one predictor effectively among several relevant predictors [9]. The failure of LASSO in this analysis might have been caused by one of two possible reasons: (1) LASSO is a penalized least-squares method with constraints on the absolute values of the regression coefficients. Due to this special penalty, LASSO leads to sparse selection of independent variables by shrinking most of the regression coefficients to zero. It might discard the most relevant coefficients. (2) The traits analyzed are controlled by polygenes, i.e., the infinitesimal model applies to the traits. EN regularization does both ridge and LASSO penalties simultaneously; thus, the result of the EN penalty is a combination of the effects of the two methods [5]. Because ridge penalty shrinks predictors efficiently, these coefficients are nonnull with a large number of SNPs; therefore, it was inefficient and led many estimates to vanish. LASSO penalty compresses the small coefficients to zero, which provides effective control for high-dimensional variable selection, particularly in cases in which only a small subset of SNPs with large coefficients are associated with the trait. By combining ridge and LASSO penalties, EN can average markers that are highly related with a trait and then the averaged marker is entered into the model. Thus, EN is an adjustable model to fit traits with different genetic structures. Our results provide guidance for choosing the optimal parameter α.

### Comparison of EN, GBLUP and BayesB models to estimate GEBV in two validation procedures

The GBLUP method, which assumes a normal distribution of marker effects with an equal variance and that all SNPs have a nonnull effect, performed better than the BayesB method for each trait. Many studies show that the GBLUP model performs well for most polygenic traits in livestock [8,24]. The worst performance of BayesB for most traits in our study is not particularly notable. Previous studies have reported that the accuracy of BayesB differs among traits, which depends on the genetic architecture of the traits [8]. Bayesian methods divide the SNPs into two parts, in which a high proportion SNPs have null effect (π), while other SNPs have large or moderate effects. Therefore, the performance is better for traits controlled by a few QTLs. We also fixed π = 0.99 and 0.999 in the BayesB method for the CW, ADG, EMA, and MS traits (Table 5), but the results were worse than those at π = 0.9 for each trait. These results suggested that four traits are controlled by many SNPs, especially ADG and MS. Previous studies suggest a value for π, but the challenge remains to identify the most accurate π in a BayesB model. Other challenges were computational speed and memory consumption. BayesB had no computational advantage, requiring 1.4 days for a prediction. With GBLUP, more time (32 min) was required to construct the genomic matrix, but the average total time for GBLUP (32.26 min) was approximately 20 min less than that with the EN method with alpha = 0.001 (52.4 min). However, we were only required to compute the genomic matrix once for a common population. Thus, GBLUP had the absolute advantage in computing time.

In the comparison of the EN with BayesB and GBLUP, the EN model was superior to the BayesB and GBLUP for most traits. The computational speed of EN was between those of GBLUP and BayesB. EN is a machine learning method that uses training sets to learn parameters in models and then predict the test sets with these models. Unlike GBLUP, which only outperformed in traits controlled by large SNPs with small effects, EN performed well in two models (the infinitesimal model and finite model) if a suitable alpha was set. Since the EN method provided higher prediction accuracy for six of ten traits and because of its adjustability, it was a good choice for further GS in Chinese Simmental beef cattle.

### Comparison of the traits analyzed in two validation procedures

GS in Chinese Simmental beef cattle has been evaluated since 2008, but to date, GS has been not implemented in practical breeding. The reference population has been continuously updated, although with a limited increase in number. Many factors hamper the development of GS in China. A comparison of our results from the CV procedure with those in other beef cattle (Table 2) shows that [25] reported a prediction accuracy of MS lower than that for CW and EMS but with a higher heritability in Hanwoo beef cattle, which was the same result as in our study. For the CW trait, other studies show that the accuracy of the Bayesian method was higher than that of GBLUP and that of Simmental cattle in previous studies [11,26], which suggests that CW is controlled by QTLs with large effect. However, our study produced a different result. For the EMA trait, the accuracy for Hanwoo, Nellore, American Angus, and Japanese Black was 0.317, 0.47 (realized accuracy), 0.698, and 0.42, respectively. The highest accuracy for the EMA trait in this study was 0.287 using GBLUP, which corresponded with that of Nellore using BayesLasso. For the ADG trait, the performance of prediction differed in methods with Chinese Simmental, Nellore and multibreed cattle. The highest accuracy for the ADG trait in our study was 0.251 using EN (α = 0.001). The accuracy values for Chinese Simmental in a previous study, Nellore and multibreeds were 0.214, 0.26 and 0.24, respectively.

In contrast, when generation validation was used, the accuracies of three traits using three methods were slightly higher. This might be caused by the larger training dataset and smaller test dataset. Few reports are available about the traits TW, SW, BNW, CL, and HLL. However, clear correlations between traits were observed, and TW and SW are also of economic interest. These observations may indicate that the genetic architecture of these traits is different among breeds, which could cause the different characteristics in different breeds of cattle. We suggest that ADG, CW, EMA and MS traits are suitable for the infinitesimal model, as these traits have small effects distributed on many loci.

The accuracy of genomic predictions is influenced by the density of the SNP panel, the heritability of the trait, the training population size and the effective population size [27]. In this study, the estimated effective population size five generations ago (*N*_*e*_ = 103) was consistent with that reported by [11]. The estimates of heritability were moderate to high but with low to moderate prediction accuracies. One of the main reasons for the limited accuracies, ranging from 0.17 to 0.295, could be due to the small reference population size (*N* ≈ 973). Another influence factor is CV strategy to assess the predictive ability of different methods. There are already a few studies that show that a stronger genetic relationship between the training and test datasets results in the highest accuracy for GEBV [28,29]. The averages of the relationship between two datasets in each validation procedure ranged from 0.08 to 0.09. This result indicates that the level of relationship between the training and validation populations was relatively low, which may have affected the accuracy. To improve the accuracy of the genomic prediction in this population, the key point would be to increase the number of reference animals. Considering the cost of genotyping, we can use lower density panels (80 K or 50 K). Another alternative method is to obtain the pedigree information and use this information simultaneously with genotype information in one model, such as single-step GBLUP (ssGBLUP). Therefore, the use of genomic prediction in real breeding programs for Chinese Simmental cattle is far off since there is no reasonable reference population.

## Conclusions

The performance of the statistical methods used depended on the trait analyzed. The results showed that GBLUP and EN were clearly superior to BayesB for each trait, particularly LW and CL. When an appropriate alpha was set in the EN model, the results were better than those of GBLUP for most traits, with the exception of traits EMA, CL and MS. EN and GBLUP methods could be equally recommended for the implementation of GS for carcass traits in Chinese Simmental cattle. In addition, the results also suggest that the genetic architecture underlying the ADG, CW, EMA and MS traits are similar and are controlled by many SNPs. Overall, our results can be used as a reference for implementing genomic prediction in Chinese Simmental beef cattle.

## Supporting information

S1 Fig Kinship plot for all individuals. (PNG)

S1 Table Genotypic (below diagonal) and phenotypic (above diagonal) correlations and standard errors (SE) between all pairs of ten traits. (DOCX)
