# Supplementary figures and images for: Evaluation of GBLUP, BayesB and elastic net for genomic prediction in Chinese Simmental beef cattle
Source: PLoS One. 2019 Feb 28;14(2):e0210442. doi: 10.1371/journal.pone.0210442 (PMC6394919; doi:10.1371/journal.pone.0210442)

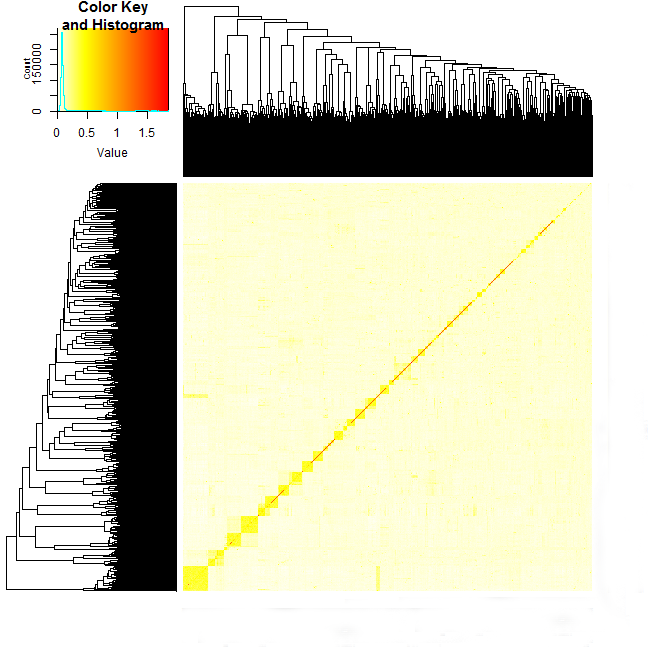

Supplement: S1 Fig — (PNG) [file pone.0210442.s001.png]
