# Supplementary material for: Evaluation of GBLUP, BayesB and elastic net for genomic prediction in Chinese Simmental beef cattle
Source: PLoS One. 2019 Feb 28;14(2):e0210442. doi: 10.1371/journal.pone.0210442 (PMC6394919; doi:10.1371/journal.pone.0210442)
Supplement: S1 Table — (DOCX) [file pone.0210442.s002.docx]

S1 Table

Supporting text to

Evaluation of GBLUP, BayesB and Elastic Net for Genomic Prediction in Chinese Simmental Beef Cattle

Xiaoqiao Wang^1,¶^, Xue Gao ^1,¶^, Jian Miao ^1^, Tianpeng Chang^1^, Jiangwei Xia^1^, Binxin An^1^, Yan Li^2^, Lingyang Xu^1^, Lupei Zhang^1^, Junya Li^1,*^, and Huijiang Gao^1,*^

^1^ Laboratory of Molecular Biology and Bovine Breeding, Institute of Animal Sciences, Chinese Academy of Agricultural Sciences, Beijing, 100193, China

^2^ Veterinary Bureau of Wulagai Precinct in Xilin Gol League, Wulagai, China

**S1 Table Genotypic (below diagonal) and phenotypic (above diagonal) correlations and standard errors (SE) between all pairs of ten traits**

Trait: ADG average daily weight gain, LW live weight, CW carcass weight, BNW bone weight, SW sirloin weight, TW tenderloin weight, EMA eye muscle area, CL carcass length, HLL hand legs length, MS marbling score

| Trait | ADG | LW | CW | SW | TW | EMA | CL | HLL | MS | BNW |
| --- | --- | --- | --- | --- | --- | --- | --- | --- | --- | --- |
| ADG | 1 | 0.89 ± 0.06 | 0.78 ± 0.01 | 0.57 ± 0.03 | 0.58 ± 0.04 | 0.08 ± 0.07 | 0.47 ± 0.04 | 0.42 ± 0.04 | 0.10 ± 0.05 | 0.52 ± 0.04 |
| LW | 0.97 ± 0.01 | 1 | 0.92 ± 0.01 | 0.58 ± 0.02 | 0.60 ± 0.03 | 0.45 ± 0.04 | 0.50 ± 0.02 | 0.43 ± 0.02 | -0.05 ± 0.03 | 0.55 ± 0.02 |
| CW | 0.88 ± 0.04 | 0.97 ± 0.02 | 1 | 0.54 ± 0.03 | 0.63 ± 0.02 | 0.51 ± 0.04 | 0.52 ± 0.04 | 0.48 ± 0.04 | 0.14 ± 0.05 | 0.55 ± 0.02 |
| SW | 0.71 ± 0.10 | 0.36 ± 0.1 | 0.50 ± 0.15 | 1 | 0.52 ± 0.04 | 0.51 ± 0.04 | 0.40 ± 0.04 | 0.32 ± 0.04 | 0.03 ± 0.05 | 0.42 ± 0.04 |
| TW | 0.66 ± 0.09 | 0.58 ± 0.11 | 0.95 ± 0.15 | 0.61 ± 0.08 | 1 | 0.52 ± 0.04 | 0.36 ± 0.04 | 0.33 ± 0.05 | 0.24 ± 0.05 | 0.50 ± 0.04 |
| EMA | 0.11 ± 0.12 | 0.50 ± 0.12 | 0.55 ± 0.10 | 0.76 ± 0.10 | 0.66 ± 0.09 | 1 | 0.12 ± 0.05 | 0.18 ± 0.05 | 0.18 ± 0.05 | 0.33 ± 0.05 |
| CL | 0.59 ± 0.13 | 0.71 ± 0.10 | 0.68 ± 0.11 | 0.55 ± 0.14 | 0.37 ± 0.13 | -0.02 ± 0.05 | 1 | 0.31 ± 0.05 | 0.1 ± 0.05 | 0.39 ± 0.05 |
| HLL | 0.53 ± 0.12 | 0.61 ± 0.10 | 0.62 ± 0.1 | 0.48 ± 0.13 | 0.33 ± 0.12 | 0.26 ± 0.14 | 0.59 ± 0.15 | 1 | 0.03 ± 0.05 | 0.45 ± 0.04 |
| MS | 0.60±0.18 | 0.58 ± 0.16 | 0.55 ± 0.16 | 0.45 ± 0.15 | 0.68 ± 0.15 | 0.54 ± 0.15 | 0.49 ± 0.20 | 0.09 ± 0.18 | 1 | 0.15 ± 0.05 |
| BNW | 0.68±0.1 | 0.82 ± 0.15 | 0.69 ± 0.07 | 0.56 ± 0.11 | 0.65 ± 0.09 | 0.40 ± 0.12 | 0.60 ± 0.13 | 0.72 ± 0.10 | 0.22 ± 0.16 | 1 |
